# Comparison of artefact reduction possibilities with the new active transcutaneous bone conduction implant (Bonebridge)

**DOI:** 10.1017/S0022215122000494

**Published:** 2023-03

**Authors:** S Edlinger, E Tenner, J Frühwald, G Sprinzl

**Affiliations:** 1 Department of Otorhinolaryngology, Head and Neck Surgery, University Clinic St Poelten, Austria; 2 Karl Landsteiner Institute of Implantable Hearing Devices, St Poelten, Austria; 3 Institute for Radiology, St Poelten, Austria

**Keywords:** Prostheses, Magnetic Resonance Imaging, Neuroma, Acoustic, Bone Conduction

## Abstract

**Objective:**

This study aimed to evaluate the possibilities of artefact reduction using different anatomical implant positions with the Bonebridge bone-conduction hearing implant 602 for a patient with an acoustic neuroma requiring regular diagnostic magnetic resonance imaging of the tumour position.

**Method:**

Three implant positions and magnetic resonance imaging examinations with and without customised sequences for metal artefact suppression were investigated. The diagnostic usefulness was rated by a radiologist (qualitative evaluation), and the relation between the area of artefact and the total head area was calculated (quantitative evaluation).

**Results:**

Following the qualitative analysis, the radiologist rated the superior to middle fossa implant placement significantly better for diagnostic purposes, which is in agreement with the calculated artefact ratio (*p <* 0.0001). The customised slice-encoding metal artifact correction view-angle tilting metal artifact reduction technique sequences significantly decreased the relative artefact area between 5.13 per cent and 25.02 per cent. The smallest mean artefact diameter was found for the superior to middle fossa position with 6.80 ± 1.30 cm (range: 5.42–9.74 cm; reduction of 18.65 per cent).

**Conclusion:**

The application of artefact reduction sequencing and special anatomical implant positioning allows regular magnetic resonance imaging in patients with the bone-conduction hearing implant 602 without sacrificing diagnostic imaging quality for tumour diagnosis.

## Introduction

Acoustic neuromas are benign tumours that may develop on the hearing and balance nerves near the inner ear which, depending on size, affects the hearing of the patient. The incidence has been estimated to be between 7 and 15 people per million and constitutes about 80 per cent of all tumours found in the cerebellopontine angle.^1–3^ Patients diagnosed with an acoustic neuroma can potentially be faced with three management options: observation, or the active treatments of microsurgery or radiation. Especially for the above-mentioned therapeutic options, regular observation of the status or growth of the tumour is required. Magnetic resonance imaging (MRI) is a technique that allows detection of tumors at much smaller sizes than previously possible using computed tomography (CT) or auditory brainstem responses. This imaging technique visualises internal structures of the body using magnetic and electromagnetic fields, which may cause artefacts or other problems with active implantable devices because of interferences from the internal magnet or metal.

Unlike conventional radiography and CT scanning, MRI examinations do not expose patients to ionising radiation (Organisation for Economic Co-operation and Development (2021), MRI units (indicator); accessed on 9 February 2021). Therefore, MRI has become an increasingly important and widely used diagnostic tool in clinical routine. Todt *et al*. estimated that 50 to 75 per cent of pacemaker wearers would need to have an MRI during their lifetime.^4^ Accordingly, this means that hearing implant users with an estimated implant lifetime of at least 20 years have an almost 100 per cent chance that they would need an MRI at least once during the implant lifetime period. This number increases dramatically for hearing implant users who, for example, suffer from neurofibromatosis type 2 or other tumour conditions such as vestibular schwannoma or acoustic neuroma. If, because of tumour growth or radiation therapy, hearing is affected beyond the possibility of conventional hearing aid treatment and reconstruction surgery (including partial and total middle-ear prostheses), implantable hearing devices may be applicable. Besides cochlear implants, which are indicated for patients with profound hearing loss or deafness, bone-conduction hearing implants and active middle-ear implants may effectively rehabilitate mild-to-moderate or mild-to-severe hearing losses. Based on the presented patient's hearing loss, bone-conduction devices are further exploited.

All available bone-conduction hearing implant systems are characterised by excellent sound-transmission properties of the skull bone. Sound is picked up by the externally worn audio-processor microphones and converted into vibratory stimuli that are either applied directly to the bone (‘direct-drive’ percutaneous: Baha^TM^, Ponto^TM^ or ‘direct-drive’ transcutaneous: Bonebridge (Med-El, Innsbruck, Austria) or indirectly via the skin (‘skin-drive’ transcutaneous: Sophono^TM^ and Baha® Attract).^5^ Both device categories contain magnetic and electrically conductive materials inducing difficulties when undergoing MRI measurements or even hindering diagnosis if artefacts occur within the area of interest. An artefact sphere of 15 cm in diameter was reported with Bonebridge (bone-conduction hearing implant 601),^6^ a distance of 5–10 cm was reported with the Sophono^TM^ implant^7^ and 11.5 cm was reported from the centre of the Baha Attract implant.^6–8^

Most hearing implant manufacturers nowadays make their products MRI conditional^9–11^ and allow temporal explantations of the magnet for diagnostic purposes.^12^ However, this implies two surgical procedures for the patient: one to explant and one to re-implant the magnet, during which time the implant cannot be used, resulting in ‘no hearing’ for the patient.^6–8^ Besides the effects of the MRI and the potential explantation for easier access, the main disadvantages of percutaneous systems, such as the Baha are related to its abutment and include skin reaction, wound infection, growth of skin over the abutment and implant extrusion with major complications in up to 37 per cent of the infant cases.^13^ However, even with a simplified surgical technique using a linear incision, extrusion rates have been reported in 9.3 per cent of cases.^13^

The motivation for these experiments was a patient with an acoustic neuroma presenting to the clinic with mild-to-moderate mixed and conductive hearing loss. The hearing loss resulted from an incomplete tumour resection because of the intra-operative association of the tumour with a potential facial nerve injury by the neurosurgeon. The patient showed stable disease without tumour growth for two years, but she suffered considerably from the negative impact on quality of life and showed mild signs of depression following the untreated hearing loss. After two years of unsuccessful conventional hearing aid trials, she insisted on an implantable solution. The possible rehabilitation options for her given indication (transcutaneous *vs* percutaneous systems) were discussed with the hospital implant board which recommended, for both audiological and wearing-comfort reasons, the direct-drive options that were then presented to the patient.

Following thorough explanations and counselling regarding possible artefacts that may hinder her diagnostic acoustic neuroma management, the patient decided to get the active transcutaneous bone conduction implant (Bonebridge, bone-conduction hearing implant 602, Med-El, Innsbruck, Austria). Despite being MRI conditional up to 1.5 Tesla as stated above, the reported artefact size of the precursor model, the bone-conduction hearing implant 601, is approximately 15 cm around the implant side and may also be present on the images on the contralateral side of the head.^14,15^ Utrilla *et al*. also reported substantially reduced artefact size (almost 50 per cent compared with the previous generation) with the new generation bone-conduction hearing implant 602, especially when metal artefact reduction sequences were applied^14^ (from now on also referred to as reduction sequences). The imaging artefacts do not only depend on the type of implant and the scanning parameters but also on the position of the implant in relation to the site of interest. Therefore, it is not only MRI safety for hearing implants that is essential but also the possibility to reduce the implant shadow and artefact region to allow for comprehensive clarification of diagnosis.

The aim of this study was to evaluate the possible correlation of customised metal artefact reduction sequences with three different anatomical implant positions on artefact size in a cadaver head implanted with the newest generation of active transcutaneous bone conduction implant.

## Materials and methods

### System description and imaging conditionality

The latest generation of active bone conduction implants, the bone-conduction hearing implant 602 was used. It addresses the most reported disadvantage of its precursor model, the bone-conduction hearing implant 601, which was the size of the implanted bone conduction floating mass transducer. The new generation presents with almost half the size of the previous generation, allowing implantation with a drilling depth of 4.5 mm (equal to the drilling depth of a Baha screw), which makes pre-surgical planning redundant and allows for more individual positioning options, as investigated in this study. The bone-conduction hearing implant 602 device used was provided by the manufacturer (Med-El, Innsbruck, Austria). The bone-conduction hearing implant 602 is MRI conditional at 1.5 Tesla without the need to surgically remove the magnet. The MRI scanner was limited to ‘normal operating mode’ (whole body averaged specific absorption rate of less than 2 W/kg); ‘first level controlled operating mode’ was avoided.

### Specimen preparation

**Fig. 1.**
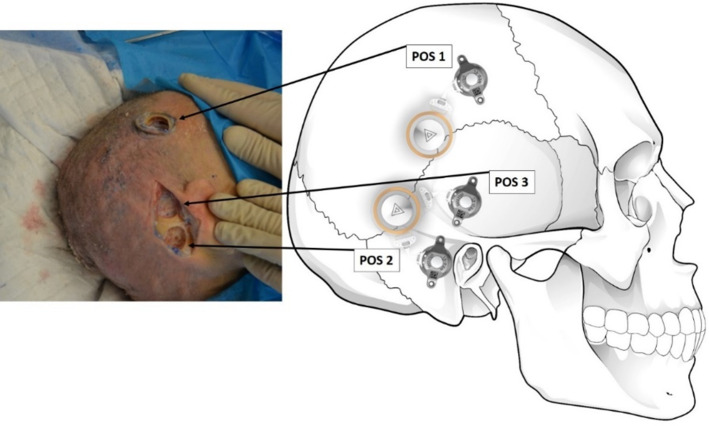
Experimental positioning of the bone-conduction hearing implant 602 device. Position 1: superior to middle fossa; position 2: classical sinodural angle; position 3: classical middle fossa. The left side of the figure shows a cadaver head with the bone conduction floating mass transducer implant bed drilled, and the right shows a schematic presentation of the bone conduction floating mass transducer and coil position, allowing for planned audio-processor placement. Pos = position

One fresh frozen cadaver head was obtained from the Institute of Anatomy, Medical University of Vienna, Austria. The fresh frozen condition enabled surgical preparation under conditions close to the intravital situation. Three different anatomical positions were prepared as indicated in Figure 1: superior to the middle fossa position, the classical sinodural angle position and the classical middle fossa position, and emphasis was placed on coil position for post-operative auditory processor application.

### Magnetic resonance imaging measurements

**Fig. 2.**
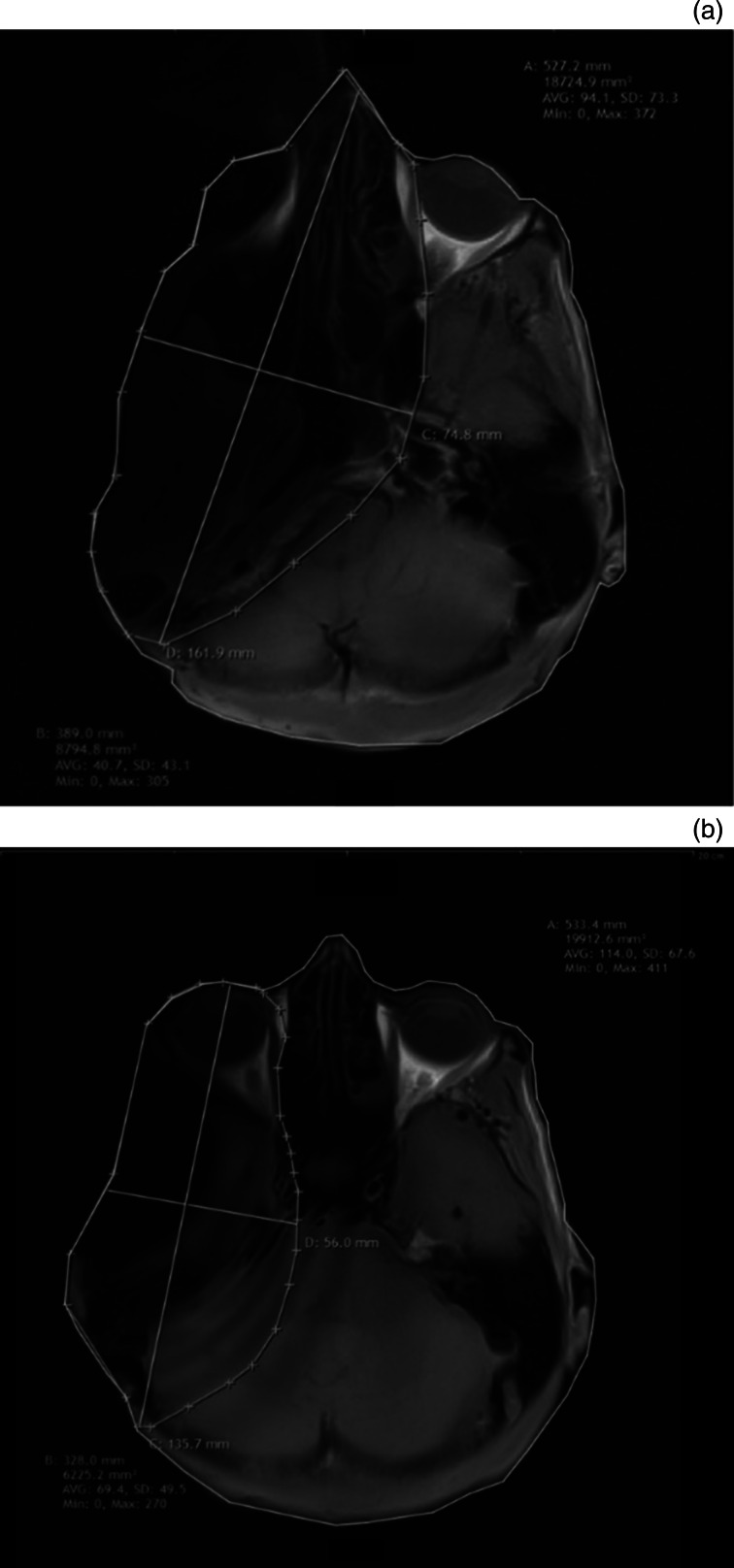
T1-weighted axial magnetic resonance imaging scans showing: (a) image without reduction sequences and (b) image with slice-encoding metal artifact correction view-angle tilting metal artifact reduction technique sequences applied. *A = measurement for the full head; B = measurement taken for area of the implant;* C/D = measurement taken for width; AVG = average; SD = standard deviation; Min = minimum; Max = maximum

The cadaver head was supine positioned without the need of additional fixation in the MRI scanner according to the standard position in routine clinical practice. All scans were obtained in a commercially available 1.5 Tesla MRI scanner applying different specifications (Siemens® 1.5 Tesla Allegra MRI scanner; Table 1). Digital imaging and communications in medicine (‘Dicom’) data from the MRI series of the patients were retrieved from the picture archiving system and transferred to a computer, which contained the Synedra Aim 15 ‘Odysseus’ software (version 15.0.0.3 × 64 edition). The Synedra software is available as a freeware from Synedra Information Technologies (Innsbruck, Austria). This software contains tools for measuring and defining distances and volume values. Using this software, artefacts around each implant were compared with the full head image radius measurement in different series (axial T1-weighted, axial T2-weighted, coronal T1-weighted and coronal T2-weighted; Figure 2).

**Table 1.** Scan parameters

Scan type	TR & TE (ms)	Flip angle (°)	Slice thickness (mm)	Matrix (pixel)
Axial T1-weighted without artefact reduction	TR 490, TE 8.7	150	5	320 × 280
Axial T1-weighted Semac Vat Warp	TR 650, TE 7	130	3	256 × 204
Coronal T2-weighted without Mars	TR 3500, TE 102	150	3	256 × 179
Coronal T2-weighted with Mars	TR 5300 TE 85	150	3	320 × 256
Axial T2-weighted with Mars	TR 2880 TE 85	150	3	320 × 256

Scanning parameters for the T1- or T2-weighted measurements applied on every anatomical position. TR = repetition time; TE = time to echo; Semac Vat Warp = slice-encoding metal artifact correction view-angle tilting metal artifact reduction technique sequences; Mars = metal artefact reduction sequences

### Imaging evaluation

**Fig. 3.**
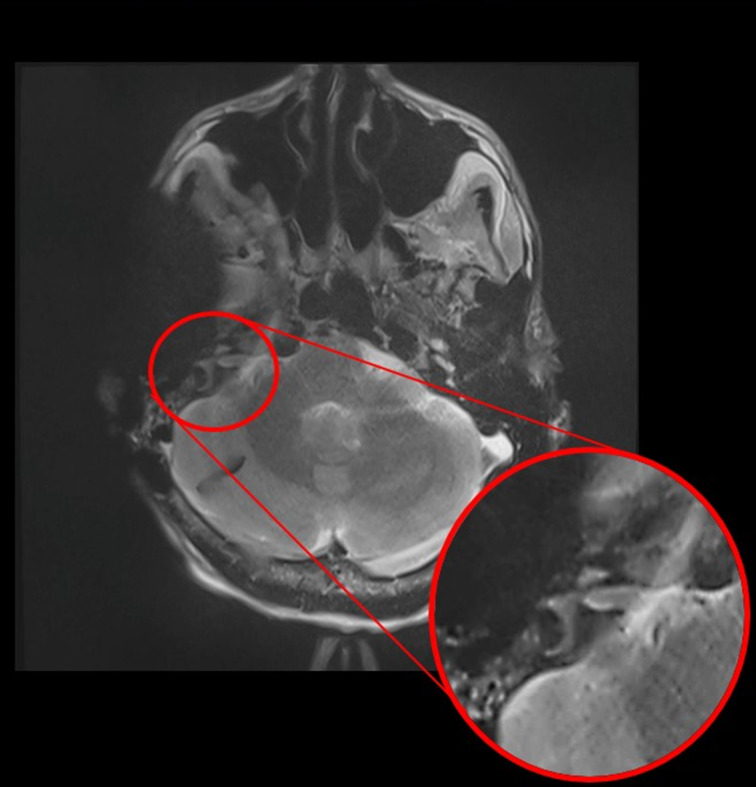
Position 1 T2-weighted axial magnetic resonance imaging scan with slice-encoding metal artifact correction view-angle tilting metal artifact reduction technique sequences. Visualisation of the brain adjacent to the artefact of the bone-conduction hearing implant 602 device with a zoom-in of the area of interest: the cerebellopontine angle and internal auditory canal are fully visible.

The artefact surrounding the implant was evaluated both qualitatively and quantitatively. For quantitative purposes, we calculated the percentage ratio of the full head area to the area of the artefact in all measured sections (Figure 2). The diagnostic usefulness and qualitative image analysis of the acquired MRI scans were rated by a consultant radiologist (Figure 3). Focus was put on the visualisation of the brain, the cerebellopontine angle and internal auditory canal adjacent to the artefact. The ability to visualise the internal auditory meatus and cerebellopontine angle cistern for the sides ipsilateral and contralateral to the bone-conduction hearing implant 602 was assessed for the measurement series axial T1-weighted, axial T2-weighted, coronal T1-weighted and coronal T2-weighted images for all three anatomical implant positions.

## Results

The study compared possible MRI artefact reduction possibilities by investigating different implant positions as well as applying metal artefact reduction sequences. The image acquisition time was 12 minutes and 20 seconds for the T1-weighted MRI and 12 minutes and 12 seconds for the T2-weighted MRI. Globally, the artefact related to the bone-conduction hearing implant 602 was less prominent in the axial plane than in the coronal plane. Furthermore, only the axial plane allowed for the visualisation of the brain parenchyma, the cerebellopontine angle and the internal auditory canal, and therefore the focus of the evaluation was directed towards the axial plane measurements. The ratio of full head to artefact size compared with no reduction sequences was investigated for T1- and T2-weighted scans in the axial plane.

**Fig. 4.**
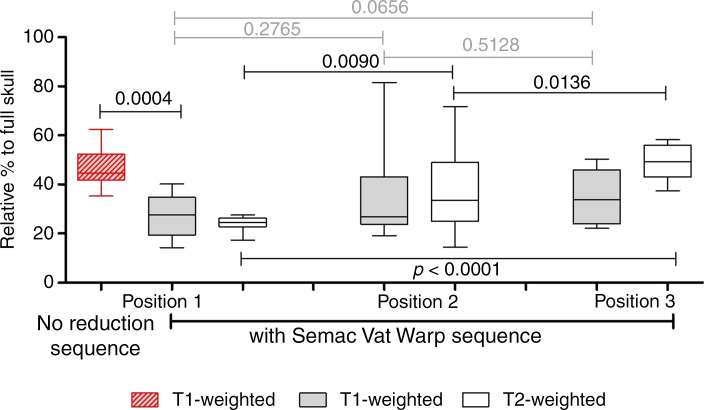
Box-plot showing the axial T1-weighted magnetic resonance imaging relative area of artefact to head percentages for the three placement positions. Position 1: superior to middle fossa; position 2: classical sinodural angle; position 3: classical middle fossa. Ends of the box are the upper and lower quartiles, so the box spans the interquartile range. The median is marked by the horizontal line inside the box.

The customised slice-encoding metal artifact correction view-angle tilting metal artifact reduction technique sequences significantly decreased the relative artefact area (percentage) in the T1- and T2-weighted sequences for the measured positions (Figures 3 and 4). In the T1-weighted sequence, the experimental superior to middle fossa position reduced mean artefact size by 42.5 per cent (*p =* 0.0004; Table 2), and the artefact reduction difference was 25.02 per cent for the classical middle fossa position and 24.02 per cent for the sinodural angle position compared with no reduction (*p =* 0.0249, *p =* 0.0214; Table 2). No significant difference was found between the three positions when applying slice-encoding metal artifact correction view-angle tilting metal artifact reduction technique sequences. Investigating the diameter of the artefact in T1-weighted sequence, the slice-encoding metal artifact correction view-angle tilting metal artifact reduction sequence reduced the mean artefact width in position 1 from 7.53 ± 1.02 cm (range: 5.90–9.07 cm) to 6.80 ± 1.30 cm (range: 4.71–9.02 cm), which translates into a 9.71 per cent reduction in size. Position 2 showed a mean artefact width of 7.14 ± 1.86 cm (range: 5.05–11.66 cm; reduction of 5.13 per cent) and position 3 showed a mean artefact width of 6.92 ± 1.65 cm (range: 4.67–9.81 cm; reduction of 8.05 per cent) (Figure 1).

**Table 2.** Outcomes for T1- and T2-weighted areas for the three tested implant positions

Parameter	T1-weighted area (mm^2^)				T2-weighted area (mm^2^)			
	No Mars	Semac Vat Warp			No Mars	Semac Vat Warp		
	Position 1	Position 1	Position 2	Position 3	Position 1	Position 1	Position 2	Position 3
Minimum	35.26	14.32	19.05	22.25	35.26	17.50	14.57	37.53
Maximum	62.57	40.23	81.53	50.34	62.57	27.50	71.76	58.23
Median	44.72	27.67	26.79	34.00	44.72	24.61	33.54	49.33
Mean	46.72	26.84	35.50	35.03	46.72	24.00	36.68	49.12
SD	8.379	8.789	18.29	10.29	8.379	2.925	16.38	7.197
*P*-value	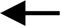 0.0004 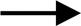		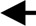 0.5128 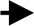		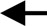 *p* < 0.0001 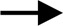		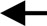 0.0136 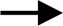	
	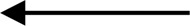 0.0214 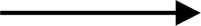				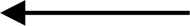 0.0809 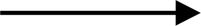			
	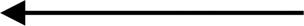 0.0249 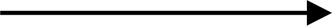				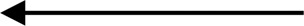 0.4316 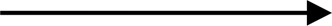			
		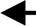 0.2765 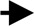				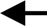 0.009 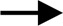		
		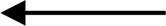 0.0656 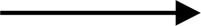				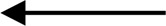 *p* < 0.0001 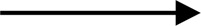		

Position 1: superior to middle fossa; position 2: sinodural angle; position 3: classical middle fossa, with and without metal artefact reduction sequences (Semac Vat Warp). Mars = metal artefact reduction sequences; Semac Vat Warp = slice-encoding metal artifact correction view-angle tilting metal artifact reduction technique sequences; SD = standard deviation

In the T2-weighted measurement, the relative artefact area (percentage) in the superior to middle fossa position with slice-encoding metal artifact correction view-angle tilting metal artifact reduction technique sequences was significantly reduced compared with no reduction sequences (*p <* 0.0001) as well as compared with the two classical approaches of middle fossa (*p <* 0.0001) and sinodural angle (*p =* 0.009). The two classical approaches significantly differed from each other (*p =* 0.0136) but were not significantly different when compared with the no reduction sequences applied. Investigating the diameter of the artefact in the T2-weighted area, the slice-encoding metal artifact correction view-angle tilting metal artifact reduction technique sequence reduced the mean artefact width in the superior to middle fossa position from 7.53 ± 1.02 cm (range: 5.90–9.07 cm) to 6.80 ± 1.30 cm (range: 5.42–9.74 cm), which translates to a reduction of 18.65 per cent in artefact width. The classical sinodural angle position exhibited a mean artefact width of 7.20 ± 1.76 cm (range: 3.89–10.48 cm), and in the classical middle fossa position a mean artefact width of 8.45 ± 1.06 cm (range: 6.51–9.91 cm) was found (Figure 1).

**Fig. 5.**
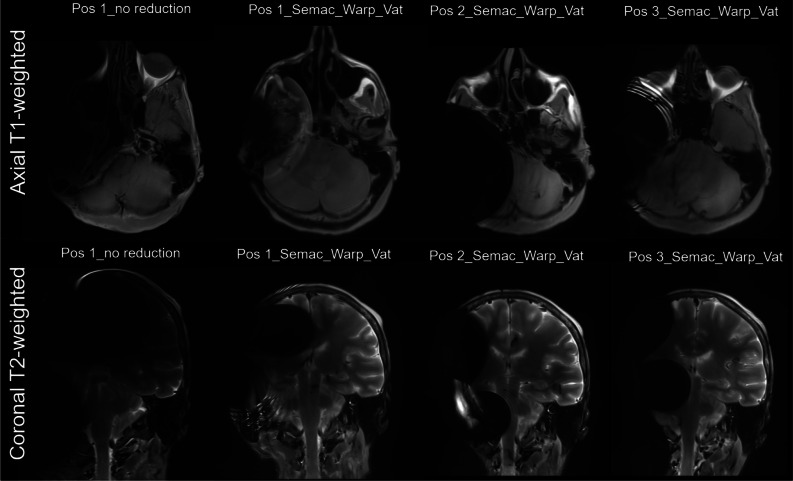
Representative visualisation of artefacts in axial plane/T1-weighted (top row) and coronal plane/T2-weighted (bottom row) without and with SEMAC-VAT WARP Metal Artefact Reduction Sequences (mars) for the 3 different implant placements. Headers indicate the implant position (Pos 1–3) and the measurement with no reduction or with MARS.

The qualitative analysis showed that the superior to middle fossa position approach allowed for a better evaluation of the cerebellopontine angle and internal auditory canal, with similar accuracy in the evaluation of the brain parenchyma (Figure 5). When artefact reduction sequences were applied, less artefact and better evaluation for both the brain parenchyma and cerebellopontine angle, and internal auditory canal evaluation was possible.

## Discussion

The aim of this study was to evaluate the possible correlation of customised metal artefact reduction sequences with different anatomical implant positions on artefact size in a cadaver head implanted with the newest generation of active transcutaneous bone conduction implants.

The major aim was to visualise the internal auditory meatus and cerebellopontine angle cistern for the sides ipsilateral and contralateral to bone-conduction hearing implant 602 for the purpose of regular diagnostics in an acoustic neuroma case without the need for implant or implant magnet removal. The motivation for these experiments was a patient with an acoustic neuroma who required regular MRI diagnostics of the respective position. This study suggested that patients diagnosed with acoustic neuroma and an active hearing implant can still undergo regular MRI examinations for routine diagnostic clarification of tumour growth.

For counselling purposes of the patient, an internal literature screening on MRI with all bone-conduction hearing implants was performed and resulted in 34 studies with a total of 440 patients out of which 215 underwent 368 scans in total.^11,16–30^ Of the 215 patients with MRI scans, data of 82 patients were from Med-El, data of 70 patients were from Cochlear Limited, 22 were from Advanced Bionics, 4 were from Oticon, 6 were from the Technical University of Vienna, 11 were from Soundtec and 1 was from Xomed. None of the extracted studies reported pain, discomfort or abortion of the scans with the Bonebridge bone-conduction hearing implant 601 device, which is the precursor model of the implant investigated here.

No cadaver studies with the sole focus on the clinical application of MRI with bone-conduction hearing implant 602 generation have been published yet.^14^ This was also one of the limitations of the present study: clinical issues such as demagnetisation, discomfort, pain or even movement of the implant during MRI testing could not be measured. The study by Utrilla *et al*. investigated both implant generations in cadaver specimens and found similar improvements in artefact reduction when applying another type of artefact reduction sequences, the so-called multi-acquisition variable-resonance image combination.

The authors also concluded that for their research the middle fossa approach allowed for better visualisation of respective brain structures with both implant versions, but the effect was more prominent with the bone-conduction hearing implant 602 device.^14^ Also worth mentioning is the fact that different surgical approaches have been described for Bonebridge implantation. The most widely used is the sinodural or mastoid placement,^15^ followed by the retrosigmoid^13^ and the middle fossa approach.^15,31^ The rather experimental superior to the middle fossa position of the bone conduction floating mass transducer was performed with emphasis on a beneficial coil position and almost similar to the classical audio-processor position. The rounded and smoothened form and the substantially reduced size of the bone conduction floating mass transducer in the new generation bone-conduction hearing implant 602 device is neither expected to have a negative effect on the patients’ hearing impression nor alters the known wearing comfort despite its relatively superior position on the skull. However, we have no experience in this matter, and we were not able to find research or publication on this subject. Importantly though, no audiological differences in those implant positions were found but similar low rates of complications and surgical times were reported.^15^ The superior to classical middle fossa position had not been used or published up to the conduct of the present study to the best knowledge of the authors. Especially for this placement, to ensure comfortable, aesthetic and beneficial audio-processor placement, emphasis was placed on an as low as possible coil position for the audio-processor, and only the bone conduction floating mass transducer was placed higher than usual.

- Acoustic neuroma incidence is estimated to be 7–15 people per million
- About 80 per cent of those tumours are found in the cerebellopontine angle
- Magnetic resonance imaging (MRI) is the ‘gold-standard’ for tumour detection, but implant artefacts may hinder regular diagnosis of possible tumour growth
- Correlation of three implant positions and MRI customised metal artefact suppression sequences were investigated
- The superior to middle fossa implant position of the Bonebridge bone-conduction hearing implant 602 allows for detailed visualisation of the cerebellopontine angle

The calculated ratio of full head to artefact size in percentage compared with no reduction sequences showed that applying the customised slice-encoding metal artifact correction view-angle tilting metal artifact reduction technique sequences significantly decreased the relative artefact area in the T1-weighted measurements for all three measured positions (experimental superior to the middle fossa, the classical middle fossa and sinodural angle) compared with no reduction sequences but was especially prominent in the experimental position superior to middle fossa. Similar but less significant results were seen in the T2-weighted measurement where the relative artefact area in the superior to middle fossa position with slice-encoding metal artifact correction view-angle tilting metal artifact reduction technique sequences was significantly reduced compared with no reduction sequences (*p <* 0.0001) and compared with the two classical approaches of middle fossa (*p <* 0.0001) and sinodural angle (*p =* 0.009). The two classical approaches significantly differed from each other (*p =* 0.0136) but were not significantly different when compared with the no reduction sequences applied. The results suggested that the experimental position superior to the middle fossa seems to be the most favourable placement to ensure diagnostic imaging quality, particularly on the implanted side.

## Conclusion

The experimental superior to middle fossa placement allowed for better visualisation of the brain areas (especially when affected by acoustic neuroma) when compared with the classical sinodural and classical middle fossa approaches. Imaging of intracranial and supra- and infra-tentorial brain pathologies are clinically more valuable than standard diagnostic MRI without any artefact reduction sequences. The sequence for metal artefact reduction enables 1.5 Tesla MRI in patients with the Bonebridge bone-conduction hearing implant 602 device without sacrificing diagnostic imaging quality, particularly on the implanted side.
